# Knowledge, Awareness, and Prevalence of Irritable Bowel Syndrome in the Saudi Community: A Cross-Sectional Study

**DOI:** 10.7759/cureus.67160

**Published:** 2024-08-18

**Authors:** Mukhtiar Baig, Zohair J Gazzaz, Wedyan E Alyoubi, Norah W AlMaslamani, Shada M Albaqami, Rawan A Almalki, Abdulaziz H Althikra, Sarah A Alosaimi, Khames T Alzahrani

**Affiliations:** 1 Department of Clinical Biochemistry, Faculty of Medicine Rabigh, King Abdulaziz University, Jeddah, SAU; 2 Department of Internal Medicine, Faculty of Medicine Rabigh, King Abdulaziz University, Jeddah, SAU; 3 Faculty of Medicine Rabigh, King Abdulaziz University, Jeddah, SAU; 4 Department of Family Medicine, King Fahad Specialist Hospital, Tabuk, SAU; 5 Department of Family Medicine, King Saud Hospital, Unaizah, SAU; 6 Faculty of Medicine, Taif University, Taif, SAU; 7 Department of Endodontics, King Faisal Specialist Hospital and Research Centre, Riyadh, SAU

**Keywords:** prevalence, abdominal pain, awareness, knowledge, irritable bowel syndrome

## Abstract

Background

The common chronic condition known as irritable bowel syndrome (IBS) lacks any visible anatomical, biochemical, or pathogenic cause. IBS significantly strains healthcare systems by sending a considerable number of patients to gastrointestinal clinics.

Objective

The present study investigated the knowledge, awareness, and prevalence of IBS among a sample of the Saudi community.

Methods

The current cross-sectional investigation was carried out from January 2, 2024, to March 15, 2024, using an electronically distributed questionnaire. IBM SPSS Statistics for Windows, Version 26 (Released 2019; IBM Corp., Armonk, New York, United States) was employed for statistical analysis.

Results

The study included 1,008 participants (655, 65% females and 353, 35% males). Most individuals (421, 42%) were from the age group of 18-30 years. Among participants, the prevalence of IBS was 31.8% (n=320). Regarding IBS knowledge, 42.2% (n=425) had low knowledge scores, 38.6% (n=389) had moderate knowledge scores, and only 19.2% (n=194) had high knowledge scores. The majority of respondents (886, 87.9%) believe that IBS affects QoL. Most participants (885, 87.8%) had good knowledge of the common symptoms of IBS. Additionally, 85.1% (n=858) of respondents recognized the psychological and emotional effects associated with IBS. Younger participants (under 20 years old) and single participants had significantly lower knowledge scores than their comparable groups (p<.001). Female participants had a higher percentage of high knowledge scores (13.4%) than males (5.9%) (p=.002).

Conclusion

The current study's findings showed that participants’ knowledge of IBS was inadequate. Around one-third of the participants suffered from IBS. Younger, unmarried individuals and females had different knowledge scores than their counterparts. The study's findings imply that further education and awareness campaigns are needed to improve understanding of IBS.

## Introduction

Irritable bowel syndrome (IBS) is a common chronic condition having no apparent anatomical or biochemical pathologic changes [1]. IBS is a frequent disorder of “brain-gut connection” worldwide, with prevalence rates ranging from 1.1 to 45% globally and 5 to 10% in most Western countries and China [2]. It is categorized by abdominal pain and irregular bowel movements [3]. There are various risk elements added to the process, such as chronic stress, anxiety, depression, and smoking [4]. Diet may also contribute to the onset of the symptoms [5]. Its prevalence in Arab countries ranged from 8.9% to 31.8% [6].

IBS patients significantly increase the healthcare budget because 20-50% of patients attend gastrointestinal clinics [7]. It affects women more than men, and in almost half of the instances, the onset occurs before the age of 35 [8]. IBS diagnosis is critical for preventing resource waste and raising patients' quality of life (QoL) [9]. A Chinese meta-analysis reported an 11.89% pooled IBS prevalence among students with gender, anxiety, depression, smoking, and drinking being the most strongly linked factors [10]. A Saudi study found a relatively high IBS prevalence (49.3%) among medical students [11]. According to this study, girls are twice as likely as boys to have IBS. Stress and unhealthy lifestyle choices were substantially linked to this high incidence [11]. It is worth noting that the rate is much higher among young people compared to other age groups [12].

IBS can also be affected by hereditary factors, with family ancestry having a role in the improvement of IBS in 30% of patients [13]. Individuals having IBS utilize more significant amounts of healthcare resources than other patients, which is evidenced by higher rates of unwarranted surgery, more tests, and more pharmaceutical use, in addition to more frequent physician visits [14]. A Makkah region survey reported a higher prevalence of IBS and found several dietary, medical, and demographic factors linked to the risk of IBS [15]. Sometimes, getting a diagnosis and treatment for this problem is challenging, greatly impacting their QoL.

Therefore, it is always important to know the knowledge and awareness of the population about this critical issue. Findings from such studies are always helpful in designing strategies to mitigate this problem and raising awareness among the public. Many studies have been published on this topic, but the extensive use of social media platforms has raised knowledge and awareness levels regarding several diseases. Therefore, it is better to assess general public awareness from time to time to get the recent data. The present study investigated the knowledge, awareness, and prevalence of IBS among a sample of the Saudi community.

## Materials and methods

The current cross-sectional investigation was carried out from January 2, 2024, to March 15, 2024. Ethical approval was obtained from the research ethics committee of King Abdulaziz University (Reference No. 718-23). The study objectives were stated at the outset of the questionnaire, and a consent statement was provided. It was also mentioned that filling out the questionnaire will be regarded as their consent to participate in this study. The inclusion criteria were adult Saudi residents aged 18 to 60 years. The healthcare practitioners and those who submitted uncompleted questionnaires were excluded from the study. The sample size was calculated using a Raosoft calculator (Raosoft Inc., Seattle, WA) with a 95% degree of confidence and a margin of error of 5%, and the estimated minimum sample size was 384. The sample size was inflated to improve the generalizability of the survey findings.

A self-administered web-based questionnaire was designed with the help of previously published studies [12,16]. The original questionnaire was prepared in English, then translated into Arabic, and back-translated to English to check its accuracy and comprehension. The questionnaire was tested on 40 people to check its clarity. Two faculty members performed the content and construct validity. The questionnaire was disseminated through a variety of social media platforms. The questionnaire contained various sections. The first section was related to the basic demographic information, such as age, gender, educational background, and marital status. The second part shed light on participants' IBS knowledge. Seventeen statements were utilized to evaluate the knowledge levels by three types of options “Yes,” “No,” and “I don’t know.” One point was awarded for the right and zero points for each erroneous or unanswered statement. The scoring scale had three categories: high knowledge (14-17 points), medium knowledge (11-13 points), and low knowledge (0-10 points) [12,16].

Statistical analysis

The data were analyzed using IBM SPSS Statistics for Windows, Version 26 (Released 2019; IBM Corp., Armonk, New York, United States). Each categorical variable was represented as frequency and percentage. The chi-square test was employed to explore the relationship between knowledge levels and demographic variables. p-values less than 0.05 were taken as significant.

## Results

The study involved 1008 participants (655, 65% females and 353, 35% males). About one-third (321, 31.8%) of participants were between 20 and 30 years old, one-fourth (252, 25%) were between 41 and 50, and the remaining belonged to different age groups. The highest number of individuals (417, 41.4%) in the central region, followed by the west, east, north, and south. Less than half of the participants (481, 47%) had a family history of IBS. Other sociodemographic descriptions of the participants are shown in Table 1.

**Table 1:** Sociodemographic characteristics of the study participants (n=1008) IBS: Irritable bowel syndrome

Parameters		Frequencies	Percentages
Age (years)	Less than 20	100	9.9
	20-30	321	31.8
	31-40	151	15
	41-50	252	25
	51-60	184	18.3
Gender	Male	353	35
	Female	655	65
Location	East	144	14.3
	Central	417	41.4
	North	124	12.3
	South	30	3
	West	293	29.1
Education level	No formal education	7	.7
	Primary	10	1
	Middle	24	2.4
	Secondary	166	16.5
	Diploma	77	7.6
	University	654	64.9
	Postgraduate	70	6.9
Marital status	Married	540	53.6
	Single	418	41.5
	Divorced	33	3.3
	Widowed	17	1.7
Heard of IBS	Relatives or friends	598	59.3
	Internet and social media	492	48.8
	The specialist doctor/specialized facility	250	24.8
	No, I haven't heard of that	91	9.0
	Other	81	8.0
Family history of IBS	Yes	481	47.7
	no	527	52.3
The most common symptoms of IBS	Diarrhea/constipation/abdominal cramps	856	84.9
	Change in appetite	332	32.9
	Inflammatory bowel	241	23.9
	Ulcers in the intestine	198	19.6
	Colon infection	79	7.8

**Figure 1:**
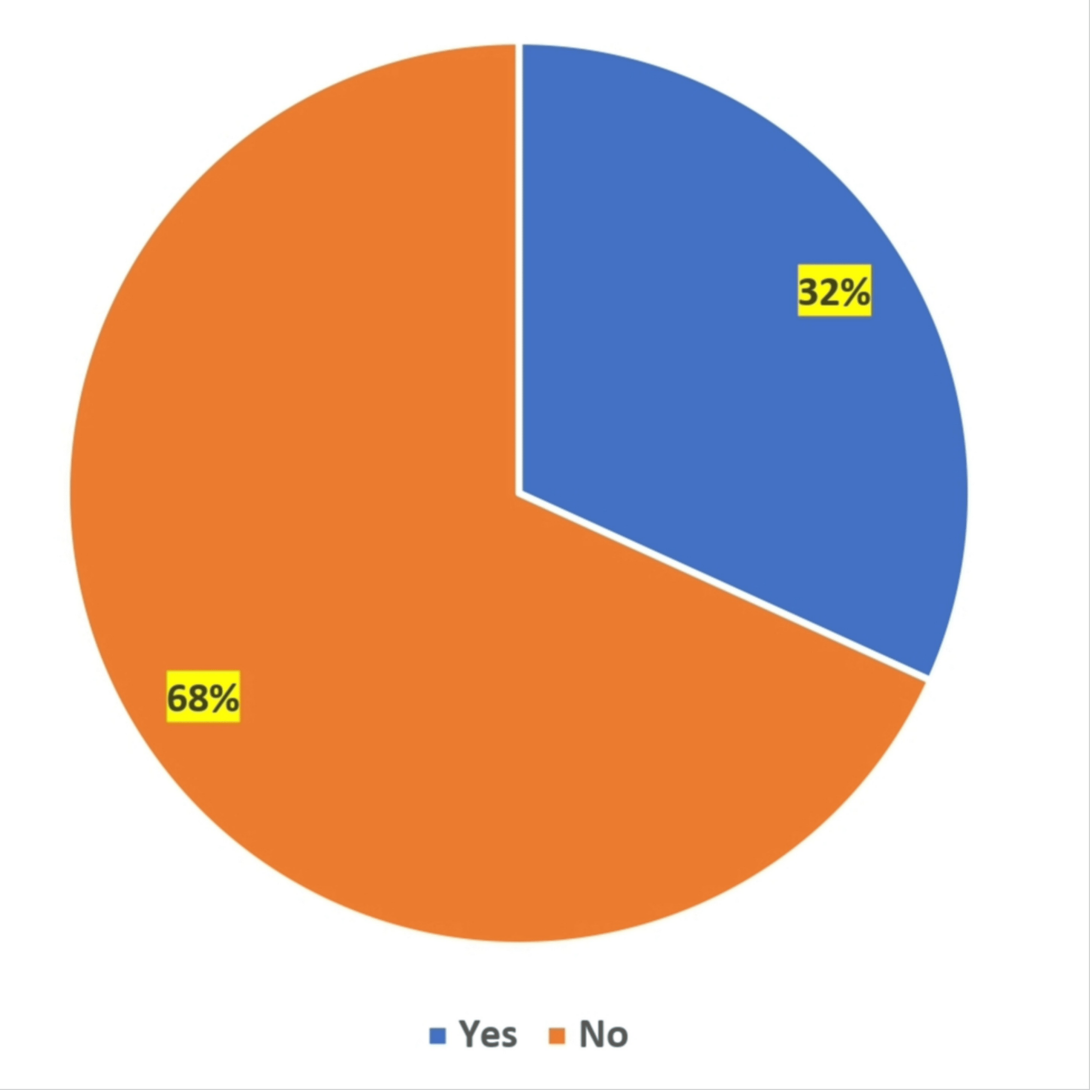
Prevalence of IBS among study participants IBS: Irritable bowel syndrome

Among participants, the prevalence of IBS was 31.8% (Figure 1). 

The majority of participants (886, 87.9%) believed that IBS affects QoL. Most participants (885, 87.8%) had high knowledge of the common symptoms of IBS. Additionally, 85.1% of respondents recognized the psychological and emotional effects associated with IBS. However, there are varying perceptions on the causes and treatment of IBS, with 46.5% attributing it to genetic factors and bacterial and viral infections and 78.6% believing that some prescription medications can improve symptoms (Table 2). 

**Table 2:** Participants’ knowledge regarding IBS (n=1008) IBS: Irritable bowel syndrome; N: number of participants, %: percentage

Statements	Yes N(%)	No N(%)	Don't know N(%)
IBS affects the quality of life	886 (87.9)	33 (3.3)	89 (8.8)
IBS is more common than diabetes and high blood pressure	362 (35.9)	207 (20.5)	439 (43.6)
Common symptoms of IBS are diarrhea, constipation, stomach pain, and flatulence	885 (87.8)	10 (1)	113 (11.2)
The most annoying symptom of IBS is abdominal pain	857 (85)	55 (5.5)	96 (9.5)
IBS can lead to hemorrhoids	436 (43.3)	90 (8.9)	482 (47.8)
IBS can lead to malnutrition	700 (69.4)	77 (7.6)	231 (22.9)
IBS can lead to absorption disorders	613 (60.8)	54 (5.4)	341 (33.8)
Colon cancer is more common than IBS	213 (21.1)	191 (18.9)	604 (59.9)
Nutritional factors, and intolerance to some foods are accompanied by an increase in the incidence of IBS	668 (66.3)	39 (3.9)	301 (29.9)
Genetic factors and bacterial and viral infections are among the most common causes of IBS	469 (46.5)	89 (8.8)	450 (44.6)
Psychological and emotional effects are disorders that are frequently associated with IBS	858 (85.1)	25 (2.5)	125 (12.4)
A change in diet may improve symptoms of IBS	838 (83.1)	32 (3.2)	138 (13.7)
Some prescription medications have a role in improving the symptoms of IBS	792 (78.6)	35 (3.5)	181 (17.9)
Alternative treatment methods (acupuncture - herbal treatment - tea - roots...) have a role in improving the symptoms of IBS	553 (54.9)	73 (7.2)	382 (37.9)
Surgical procedures can improve IBS	165 (16.4)	218 (21.6)	625 (62)
IBS is diagnosed based on symptoms:	714 (70.8)	73 (7.2)	221 (21.9)
Differential diagnosis (the sum of possible medical diagnoses for a specific disease or symptom) helps in quickly diagnosing the disease	603 (59.8)	37 (3.7)	368 (36.5)

**Figure 2:**
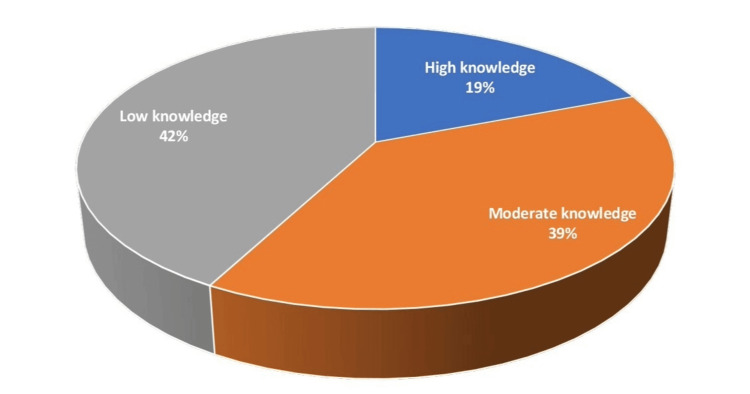
Participants’ knowledge levels

The current study found that 58% of the participants had high to moderate knowledge scores of IBS, while 42.2% had low knowledge scores (Figure 2). 

Younger participants (under 20 years old) had significantly lower knowledge scores compared to other age groups (p<.001). Single participants had significantly lower knowledge levels than other groups (p<.001). Female participants had a higher percentage of high knowledge (13.4%) than males (5.9%) (p=.002). There was no significant difference in the knowledge level according to the location (p=0.065) and educational level (p=0.384) (Table 3). 

**Table 3:** Relationship between participants’ IBS knowledge scores and their sociodemographic characteristics (n=1008) *p<0.05 was considered significant (Chi-square test), N: number of participants

Parameter		Knowledge scores			Total (N=1008)	p-value
		High knowledge	Moderate knowledge	Low knowledge		
Age	less than 20	8(0.8)	34(3.4)	58(5.8)	100(9.9)	<0.001*
	20-30	73(7.2)	108(10.7)	140(13.9)	321(31.8)	
	31-40	34(3.4)	55(5.5)	62(6.2)	151(15)	
	41-50	55(5.5)	115(11.4)	82(8.1)	252(25)	
	51-60	24(2.4)	77(7.6)	83(8.2)	184(18.3)	
Marital status	Single	74(7.3)	137(13.6)	207(20.5)	418(41.5)	<0.001*
	Married	105(10.4)	229(22.7)	206(20.4)	540(53.6)	
	Divorced	10(1)	17(1.7)	6(0.6)	33(3.3)	
	Widow	5(0.5)	6(0.6)	6(0.6)	17(1.7)	
Gender	Male	59(5.9)	119(11.8)	175(17.4)	353(35)	0.002*
	Female	135(13.4)	270(26.8)	250(24.8)	655(65)	
Location	East	26(2.6)	60(6)	58(5.8)	144(14.3)	0.065
	Middle	71(7)	173(17.2)	173(17.2)	417(41.4)	
	North	38(3.8)	47(4.7)	39(3.9)	124(12.3)	
	South	6(0.6)	11(1.1)	13(1.3)	30(3)	
	West	53(5.3)	134(13.3)	106(10.5)	293(29.1)	
Education level	No formal education	2(0.2)	3(0.3)	2(0.2)	7(0.7)	0.384
	Primary	1(0.1)	3(0.3)	6(0.6)	10(1)	
	Middle	5(0.5)	10(1)	9(0.9)	24(2.4)	
	Secondary	21(2.1)	62(6.2)	83(8.2)	166(16.5)	
	Diploma	16(1.6)	25(2.5)	36(3.6)	77(7.6)	
	University student	131(13)	261(25.9)	262(26)	654(64.9)	
	Post-graduate	18(1.8)	25(2.5)	27(2.7)	70(6.9)	

## Discussion

IBS is a global issue, and its prevalence and awareness vary in different regions. Understanding the level of IBS awareness can help healthcare professionals and policymakers develop targeted educational campaigns to increase public knowledge about IBS. This can lead to earlier recognition of symptoms and prompt seeking of medical advice, which can ultimately improve the management of the condition.

The present study showed 31.8% prevalence of IBS. Our results are similar to Egyptian and Suadi studies, which reported 27.5% and 30.5% IBS prevalence among medical students [17,18]. A Bangladeshi study reported higher IBS prevalence (39.3%) among university students [8]. The IBS prevalence rate in our study is higher than that reported in previous studies in KSA [19,20]. A recent meta-analysis reported that the pooled prevalence of IBS at KSA is 20.7% [21]. The higher prevalence of IBS among study participants could be explained by the fact that most of the study participants were younger university students. Several studies have reported that students have more stress, anxiety, and depression [22-24], and these are the contributing factors to IBS [8,17,25].

The current study found that 58% of the participants had high to moderate knowledge of IBS, and 42% had low knowledge scores. This is less than that reported in another study that evaluated university students' knowledge of IBS [26]. In that study, 74% of students knew the basics of IBS, 26% knew about its complications, 33% knew about its risk factors, 7% knew about its prognosis, and 53% knew about its treatment strategies [26]. In another study, most participants (70.5%) were aware of IBS and knew all the right information on its causes, symptoms, risk factors, prognosis, and treatment [16]. Another Saudi study reported that most participants (81.1%) had proper knowledge of IBS and its risk factors [27]. Bawahab et al. observed that more than two-thirds of the participants (70.5%) were extremely well-informed about IBS and had the correct knowledge about the causes, manifestations, risks, outcomes, and treatment [16]. Our research findings differ from a Bangladeshi study that indicated that 78% of respondents possessed inadequate knowledge of IBS [8]. According to a Saudi study, only 18.9% of the population had inadequate awareness of IBS [27].

According to our results, the majority of respondents (87.9%) believe that IBS affects QoL, with common symptoms such as diarrhea, constipation, stomach pain, and flatulence acknowledged by 87.8% of respondents. Additionally, 85.1% of respondents recognize the psychological and emotional effects associated with IBS. In a study, respondents appropriately identified symptoms of IBS, including diarrhea (48%), bloating (57%), constipation (57%), flatulence (62%), and abdominal pain (62%) [28].

Moreover, 87.9% of our study participants believed that IBS affects patients' QoL, while 70.8% reported that symptoms can diagnose it. In a previous study, two-thirds of the participants believed IBS reduces QoL, 67.9% of participants were aware IBS diagnosis is based on symptoms, and 81.2% thought medical diagnoses help quickly detect IBS [21]. It is obvious that for those who have IBS, their QoL is badly affected because of the common symptoms such as diarrhea, constipation, stomach pain, flatulence, and others. Such symptoms increase the stress level, and that further aggravates the symptoms. A vicious cycle starts in such patients who need proper management and guidance from the healthcare providers.

Raising awareness about IBS can reduce the stigma associated with the disorder. Many individuals with IBS may feel embarrassed or ashamed to discuss their symptoms, leading to delayed diagnosis and treatment. Individuals suffering from this condition may benefit by improving their awareness about the condition [27]. Such studies also provide information about the general public's misconceptions about the issue. By knowing people’s false beliefs, healthcare professionals can devise better policies to tackle the issue, and factual information can be disseminated among sufferers [13]. Besides, such studies also help in identifying particular groups in the society that need focused educational interventions and eventually improving the QoL for Saudi people affected by IBS [1].

Implications of the study

The study emphasizes the need for increased awareness about IBS among Saudis. The current study indicated that the majority of study participants' awareness regarding IBS was not up to the mark, which may contribute to delayed diagnosis and treatment. This means that there is a need for immediate action from healthcare specialists and policymakers. We recommend organizing effective public awareness campaigns and taking educational initiatives to enhance general public knowledge. People's self-awareness may assist in earlier detection and treatment of IBS and improvement in the QoL among sufferers. The present survey report emphasizes the significance of a more patient-centered approach to healthcare, in which people are empowered with knowledge and information to make informed decisions about their health. Such an approach can potentially enhance health outcomes while reducing the burden on the healthcare system.

The present survey report has several limitations. The sample size and demographic breakdown may not fully represent the Saudi population; thus, study results are not generalizable. The study data is self-reported, which could lead to reporting bias or inaccuracy. Likewise, it is possible that the study did not consider any confounding variables that could influence IBS knowledge and awareness. Future researchers should address these limitations to furnish a more comprehensive understanding of this important public health concern.

## Conclusions

The present study findings indicate that participants' knowledge of IBS was inadequate. About one-third of the participants were suffering from IBS. Younger, unmarried individuals and females had significantly different knowledge scores than their counterparts.

The study's findings propose that further education and awareness campaigns are required to improve the Saudi population's understanding of IBS. Further research and focused interventions are needed to improve IBS management and QoL in the Saudi population.
